# Investigating Cannabis-Use Among Students Attending High Schools Within the Cherokee Nation Reservation 2017 and 2019

**DOI:** 10.1007/s10900-023-01304-7

**Published:** 2023-12-08

**Authors:** Janis E. Campbell, Sixia Chen, Anna Bailey, Andrea Blair, Ashley L. Comiford

**Affiliations:** 1 Department of Biostatistics and Epidemiology, Hudson College of Public Health, University of Oklahoma Health Sciences Center, Oklahoma City, OK 73104 USA; 2 Cherokee Nation Public Health, 1325 East Boone Street, Tahlequah, OK 74464 USA; 3 Cherokee Nation Health Services, 19600 East Ross Road, Tahlequah, OK 74464 USA

**Keywords:** Cannabis use, Youth, American Indian, Health disparities, Cannabis co-use

## Abstract

Oklahoma’s medical cannabis is some of the least restrictive in the US. Previous research suggests that American Indian/Alaska Native (AIAN) have higher rates of cannabis use than other racial or ethnic groups. The goals of this paper are, first, to look at cannabis use among high school students living on the Cherokee Nation Reservation before (2017) and after (2019) medical cannabis because legal in Oklahoma (2018) utilizing the Cherokee Nation Youth Risk Behavior Survey (CNYRBS). Second, to describe the socio-demographic characteristics of youth using cannabis in the Cherokee Nation Reservation. Data were retrieved from the 2017 and 2019 CNYRBS. The data for this study included 1,216 high school students who completed the 2017 and 1,476 who completed the 2019 CNYRBS. After removal of incomplete records, there were 2,602 students whose data was analyzed in this study. Data were weighted to be representative of public-school students attending grades 9–12 within Cherokee Nation Reservation. Despite the legalization of medical cannabis in Oklahoma in 2018, there was no change in cannabis use among youth between 2017 and 2019. There were variations in cannabis use based on demographic factors and other substance uses. AIAN individuals had higher odds of current cannabis use compared to non-Hispanic White students, but there were no differences based on ethnicity. Additionally, the use of cigarettes, e-cigarettes, alcohol, and illegal drugs were associated with increased odds of cannabis use among both current and former users compared to those who had never used it. There was no spike in use among youth at least immediately after the legalization of cannabis in the Cherokee Nation Reservation. There were socio-demographic as well as substance use disparities in the use of cannabis.

**Supplementary Information:**

The online version contains supplementary material available at 10.1007/s10900-023-01304-7.

## Background

Medical cannabis is now legal in almost 75% of U.S. states, including Oklahoma where it was legalized 2018. Oklahoma is unique from many other medical legalized states because it has some of the least restrictive medical cannabis laws. It is the only state in the U.S. that does not require a qualifying condition to obtain a medical cannabis card, and minors, in restricted cases, can obtain a license. The number and proportion of Oklahomans issued a medical cannabis license is indicative of the easy access to a license. As of March 1, 2023, 6,975 growers and 2,893 dispensaries were located in Oklahoma and 368,023 Oklahomans had been issued an active medical cannabis card from the Oklahoma Medical Cannabis Authority (OMMA) [1]; close to 10% of the total population of Oklahoma. Additionally, through 2021, there were no limit on the number of dispensary licenses issued by the OMMA and costs associated with obtaining a dispensary licensing were inexpensive relative to other states at $2,500. However, in 2022, the Oklahoma state legislature increased the fees to a tiered system of between $2,500 and $10,000 depending on volume and annual sales for dispensaries. Further, in 2022, the state legislature placed a pause on new business licenses through August 1, 2024. Currently, the number of dispensaries in Oklahoma is greater than in any other state that allows medical cannabis [2]. Oklahoma has surpassed the number of dispensaries in California [3]; a significantly larger state, with over 39.2 million people compared to Oklahoma’s population of 4.0 million [2].

Medical cannabis is used to ease pain, control nausea and vomiting, and increase appetite for conditions such as cancer, HIV/AIDS, seizure disorder, glaucoma, severe chronic pain, severe nausea, severe muscle spasms, multiple sclerosis, and sleep disorders [4]. Side effects include a fast or irregular heartbeat, dizziness, slow reaction times, drowsiness, short term memory loss, trouble concentrating, and confusion [4]. The impact of cannabis use include both mental and physical health problems [5]. Longer term effects of cannabis use include mood disorders [6–8], exacerbation of psychotic disorders in vulnerable people [9, 10], self-harming [11], bullying victimization [12], cannabis use disorders [13], nicotine co-use [14, 15], withdrawal syndrome [16], neurocognitive impairments [17], cardiovascular diseases [18, 19], and respiratory diseases [20]. It is speculated that some of these conditions may be even more severe when children or youth use cannabis [21, 22].

Previous research suggests that American Indian/Alaska Native (AIAN) populations have higher rates of cannabis use. Swaim and Stanley (2018) showed that AIAN 8th, 10th, and 12th graders had higher rates of both lifetime and 30-day cannabis use compared to overall U.S. youth [23]. Published 2014 National Survey on Drug Use and Health (NSDUH) data, found that AIAN youth had the highest prevalence of cannabis use in the past month compared to other racial and ethnic groups [24]. Specifically, 22.5% of AIAN youth ages 12–17 years reported using cannabis in the past month, compared to 14.8% of White youth, 12.5% of Black youth, and 10.5% of Hispanic youth [24]. Another study using the same data (NSDUH), showed that AIAN youth have the highest cannabis use outcomes of any racial group in the U.S [25]. Among AIAN youth living near a reservation in western U.S., a 2016 study found that 42% of the participants reported using cannabis in the past year [26]. This was higher than the national average for youth of all races and ethnicities [26]. Moreover, the downstream effects of cannabis use have shown to be higher among AIAN youth [26]. Wu et al. demonstrated that AIAN youth were more likely than other racial or ethnic groups to report cannabis use disorder [27]. A study conducted in the Northern Plains showed that by age 13, 50% of AI youths had tried cannabis [28] . Stanley, Swaim [26] showed that early onset of cannabis use and alcohol intoxication is a predictor of prescription drug misuse among AI as well as non-AI youth living near a reservation.

There is conflicting evidence that legalizing medical or recreational cannabis make it easier for youth to get cannabis; thus, increasing cannabis use [29–32]. Some literature suggests a link between availability and use even controlling for perceived risk, descriptive norms, and injunctive norms related to use [33, 34]. In fact, early research (2010s) suggested that increased availability of cannabis increased cannabis use in both adults and youth [30, 35]. A 2012 study using National Epidemiologic Survey on Alcohol and Related Conditions (NESARC) and NSDUH both among adults aged 18 + , showed where states allowed medical cannabis there was higher cannabis use among adults [35]. A more recent study using the NSDUH and the Treatment Episode Dataset – Admissions (TEDS-A) datasets using a difference in differences model showed adolescent and young adult past month cannabis use increased in states that had implemented recreational cannabis [36]. Contrasting these studies are several other studies. A 2022 study by Gabri and Galanti from eleven European countries showed self-reported cannabis use among adolescence did not increase when recreational cannabis legalization [37]. A study comparing two South American countries found that where legalized recreational cannabis (Uruguay) and without legalized cannabis use (Chile) reported that cannabis use did not increase after the implementation among 8th, 10th, and 12th graders [38]. Another recent study looking across 18 U.S. states, Canada, and Uruguay (all have legalized recreational cannabis use) showed no increase in use [39]. A national study using Youth Risk Behavior Survey (YRBS) found no association in either recreational or medical cannabis legalization with current cannabis use among high school students [40]. Finally, a recent review concluded that most studies found no significant association between recreational cannabis legalization and increased prevalence of cannabis use in adolescence [32].

With the unclear evidence it is important to look at other settings, data sets, and study designs to determine if there is any link between medical cannabis availability and use and whether that holds true in a rural, highly AIAN population of youth in a reservation area. The goal of this paper is twofold. First, to look cannabis use among high school students within the Cherokee Nation Reservation before (2017) and after (2019) the law came into effect in Oklahoma (2018). Second, to describe the socio-demographic and other substance use characteristics of public-school youth in Cherokee Nation Reservation using cannabis.

## Methods

### Population

Data were retrieved from the 2017 and 2019 CNYRBS. Cherokee Nation Public Health conducts this survey biennially to better understand how risky health behaviors change over time among students attending regular public high schools located within the Cherokee Nation Reservation in Oklahoma. The sampling process and description of survey techniques have been described previously [41]. The overall response rate of this survey was 62% in 2017 and 75% in 2019. Overall, 2602 records provided valid data for the final analysis. The response rate and weighting procedure for the surveys was described elsewhere [41]. For this study we used weighted estimates as representative of all students in grades 9–12, whose reported race/ethnicity was AIAN or non-Hispanic White (NHW) or Hispanic and attending Oklahoma public schools within the Cherokee Nation Reservation for years 2017 and 2019.

### Primary Outcome

The primary outcome variable used in this analysis is from a series of questions on cannabis use from the CNYRBS (Online Resource 1). Use of cannabis was categorized as current, former, or never users. Current users were defined as using cannabis one or more times during the past 30 days. Former users were those who have used cannabis in their lifetime (no matter the number of times) but have not used in the past 30 days. Never users were those who did not report use in the last 30 days and in their lifetime.

### Sociodemographic

Survey participants were asked about age, sex, grade, and race/ethnicity. Sex was categorized as male and female. The grade variable was categorized as 9th grade, 10th grade, 11th grade, and 12th grade. The ethnicity variable was based on the following two questions: “Are you Hispanic or Latino?”; “What is your race? (Select one or more responses).” Individuals who identified as AIAN either alone or in combination with other racial groups were categorized as AIAN, regardless of how they answered the Hispanic or Latino question. Individuals who identified solely as White on the racial question and answered no to the Hispanic/Latino question were categorized as NHW; all other responses for race were categorized as Other. Age was categorized as less than 15 years, 15, 16, 17 or 18 years or older.

### Other Substance Use

The questions used in this study are listed in Online Resource 1. Cigarette use was defined as cigarette consumption one or more times during the past 30 days (current), cigarette use anytime in their lifetime (no matter the number of times) but have not used in the past 30 days (former), and those who did not report use in the last 30 days and in their lifetime (never). E-cigarette use was categorized as none in the last month or in their lifetime (never), those who have used in the last 30 days (current) and those who have used in their lifetime but not in the last 30 days (former). Smokeless cigarette users include those who reported use in at least one day in the past month (current) and those who used smokeless tobacco no days in the past month (non-user). Alcohol use one or more times during the past 30 days (current) was compared to those who reported drinking alcohol in their lifetime (no matter the number of times) but have not in the past 30 days (former) and those who did not report use in the last 30 days and in their lifetime (never). Binge drinkers were identified if they reported binge drinking alcohol one or more times during the past 30 days (current) or denied any binge drinking in the past month (non-binge drinker). Illegal drug use was categorized as none in the last month or in their lifetime if a student reported not using any of the drugs of interest (non-user) or those who have used one or more illegal drug in the last 30 days (current).

### Statistical Analysis Method

The data for this study included 1216 high school students who completed the 2017 and 1476 who completed the 2019 Cherokee Nation YRBS. After removal of incomplete records, there were 2602 students whose data was analyzed in this study. Data were weighted to be representative of public-school students attending grades 9 through 12 within the Cherokee Nation Reservation. Weighted frequency and percentages were obtained for all categorical variables. Ninety-five percent confidence intervals (95% CI) of the percentages were computed by using Taylor linearization variance estimators. Binary associations between categorical variables were examined by using the Rao-Scott Chi-square test. Design features including variance stratification, clustering, and survey final weights were incorporated into the analysis. SAS 9.4 procedure “PROC SURVEYFREQ” was used to obtain the above results. Missing values were removed from the data file for statistical analysis.

Descriptive statistics of categorical variables were compared using proportions and corresponding confidence limits. Significant differences between the two survey years were identified by a lack of overlapping confidence limits.

The SAS 9.4 procedure “PROC SURVEYLOGISTIC” was used to perform weighted logistic regression outcome to predict the influence of all collected covariates on the outcome of cannabis use as categorized as ‘Current’ vs ‘Never’ use. After generating descriptive statistics for all categorical variables, univariate modeling was conducted with all covariates of interest individually modeled against the outcome of cannabis use. Covariates with significant correlation in individual logistic regression were added to a full model with all potential interaction terms, and backward manual variable selection was used to reduce the number of predictors in the full model. Variables were dropped from the model if their significance exceeded the established alpha of 0.05. Significant interaction terms were retained in the model and are to be interpreted in the results section. Collinearity among predictors was checked using Pearson’s Correlation coefficient with a threshold of 0.70. Significant predictors with high collinearity were then checked for completeness and one of the terms would be dropped in favor of the other descriptor. Confounding was investigated by dropping each predictor from the model individually and comparing model estimates for remaining predictors. Values which had a magnitude change of more than 20% or changed directions would indicate the presence of a confounding effect from the removed variable. Odds ratios were generated for individual predictors after adjusting for the rest of the terms in the final full model.

## Results

There is no difference in terms of cannabis use between 2017 and 2019 (*p* = 0.75, Table 1). Population characteristics between the two years show no significant differences in terms of age (*p* = 0.94), grade (*p* = 0.99), sex (*p* = 0.95), race (*p* = 0.87), and ethnicity (*p* = 0.10, Table 1). We see a significant increase in the use of e-cigarettes from 2017 to 2019 (*p* = 0.038, Table 1). Finally, there was no change in 2019 from 2017 in cigarette use (*p* = 0.07), smokeless tobacco use (*p* = 0.16), alcohol use (*p* = 0.16), binge drinking (*p* = 0.34), or illegal drug use (*p* = 0.88, Table 1).

**Table 1:** Sample number, estimated population, percent and 95% confidence interval of population characteristics Cherokee Nation Youth Risk Behavior Survey Cherokee Nation Reservation 2017 and 2019

	2017				2019				p-value
	n	N	Percent N	95% CI	n	N	Percent N	95% CI	
**Age**									0.9412
14 years or younger	118	2090	8.9	3.1–14.7	114	1444	6.4	2.9–9.9	
15 years	320	5291	22.5	16.2–28.8	396	5289	23.4	16.6–30.1	
16 years	370	5958	25.3	20.4–30.2	348	5853	25.9	21.6–30.1	
17 years	255	5935	25.2	18.2–32.3	315	5566	24.6	20.5–28.7	
18 years or older	109	4249	18.1	6.0–30.1	253	4489	19.8	12.4–27.2	
**Grade**									0.9995
9th	334	6349	27.0	14.5–39.4	470	6009	26.5	16.1–36.9	
10th	436	6037	25.6	14.2–37.1	329	5773	25.5	20.6–30.4	
11th	268	5764	24.5	17.8–31.2	328	5522	24.4	18.6–30.2	
12th	135	5394	22.9	9.6–36.3	302	5369	23.7	15.0–32.4	
**Sex**									0.9517
Male	559	11,934	50.8	43.3–58.3	730	11,530	51.0	47.9–54.2	
Female	612	11,549	49.2	41.7–56.7	691	11,058	49.0	45.8–52.1	
**Race**									0.8653
American Indian Only	482	10,464	48.2	41.2–55.2	520	9703	48.9	43.4–54.4	
Non-Hispanic White	567	11,258	51.8	44.8–58.9	645	10,151	51.1	45.6–56.6	
**Ethnicity**									0.0980
Hispanic	106	1446	6.2	4.0–8.4	181	1979	8.9	6.2–11.4	
Non-Hispanic	1050	21,823	93.8	91.6–95.9	1228	20,390	91.2	88.6–93.7	
**Cannabis use**									0.7479
Never	794	15,521	67.2	62.5–72.0	925	14,461	65.4	60.2–70.7	
Former	196	4077	17.7	15.4–19.9	245	3933	17.8	14.9–20.8	
Current	163	3483	15.1	10.7–19.5	218	3714	16.8	13.2–20.4	
**Cigarette use**									0.0678
Never	686	13,403	61.2	55.9–66.6	936	14,168	65.9	61.8–69.9	
Former	276	5374	24.6	19.7–29.4	307	5188	24.1	20.7–27.5	
Current	137	3109	14.2	10.8–17.6	113	2157	10.0	8.0–12.0	
**E-cigarette use**									0.0376*
Never	611	11,401	57.7	50.4–65.0	623	9278	49.4	43.4–55.1	
Former	229	4432	22.4	17.5–27.4	253	4037	21.5	18.7–24.3	
Current	171	3928	19.9	12.7–27.1	321	5468	29.1	25.0–33.2	
**Smokeless tobacco use**									0.0773
None in Past Mo	1022	20,541	89.9	85.9–93.8	1322	20,874	93.2	91.8–94.5	
Current	122	2312	10.1	6.2–14.1	88	1531	6.8	5.5–8.2	
**Alcohol use**									0.1566
Never	378	7163	36.3	30.7–41.8	570	8473	41.0	35.2–46.8	
Former	311	5681	28.8	24.7–32.8	376	6074	29.4	26.6–32.2	
Current	313	6919	35.0	29.8–40.2	356	6116	29.6	25.1–34.1	
**Binge drinking**									0.3407
None in Past Mo	902	17,574	82.2	78.7–85.8	1146	17,849	84.5	80.8–88.3	
Current	169	3800	17.8	14.2–21.3	181	3270	15.5	11.7–19.2	
**Used illegal drugs**									0.8792
Yes	235	5081	22.3	17.6–26.9	297	4784	21.9	18.7–25.1	
No	903	17,758	77.8	73.1–82.4	1088	17,107	78.1	74.9–81.4	

^*^p value significant

Patterns of cannabis use show expected results. Current use of cannabis increased by age in both 2017 and 2019 from age 14 (and younger) to 16 years old. In 2017, the frequency of 17-year-olds currently using cannabis was higher than 16-year-olds and the opposite trend was observed in 2019 with fewer students using at age 17 than 16 (Table 2). Then, at age 18, there was a drop in frequency of current cannabis use in 2017 contrasting the increased frequency of 18-year-olds currently using cannabis in 2019 (Table 2). Similarly with grade, current use of cannabis increased steadily from 9 to 11th grade in 2017 with a drop in the 12th graders reporting current use; however, current use increased from 9 to 10th grade in 2019 with a drop in 11th graders followed by a jump in 12^th^ graders reporting current cannabis use. Overall, the highest frequency of students’ responses reflected never having used cannabis regardless of age or grade. There were no significant differences in cannabis use between males and females. While not significant, the highest frequency of current cannabis use occurred among AIAN, and there is no significant difference based on ethnicity (Table 2). Notably, the distribution of cannabis use was not significantly different by race in 2017 (*p* = 0.85) but did significantly differ by race in 2019 (*p* = 0.04).

**Table 2:** Current, former, and never cannabis use binary association by years Cherokee Nation Youth Risk Behavior Survey, Cherokee Nation Reservation 2017 and 2019

Population characteristics	2017				2019			
**Age**	Current	Former	Never	< .0001*	Current	Former	Never	0.0007*
14 years or younger	5.9 (0.0–13.9)	10.9 (6.1–15.8)	83.1 (72.2–94.1)		14.2 (8.5–19.8)	9.7 (3.1–16.2)	76.2 (67.2–85.2)	
15 years	11.2 (5.5–16.9)	12.8 (9.1–16.4)	76.0 (68.3–83.7)		13.0 (8.1–18.0)	14.6 (10.7–18.4)	72.4 (65.4–79.5)	
16 years	13.5 (8.6–18.3)	18.1 (15.0–21.3)	68.4 (61.6–75.2)		20.5 (13.9–26.9)	16.3 (12.3–20.2)	63.3 (55.4–71.1)	
17 years	23.8 (16.2–31.3)	13.2 (7.7–18.8)	63.0 (56.9–69.2)		13.4 (8.2–18.7)	19.8 (14.9–24.7)	66.8 (60.5–73.1)	
18 years or older	15.0 (10.4–19.5)	32.6 (26.7–38.5)	52.4 (45.8–59.0)		21.0 (14.4–27.6)	23.6 (16.3–30.9)	55.3 (46.1–64.6)	
**Grade**				< .0001*				0.0034*
9th	8.4 (3.1–13.7)	10.5 (7.2–13.8)	81.1 (73.9–88.3)		14.6 (10.3–18.9)	11.7 (7.1–16.2)	73.8 (65.9–81.7)	
10th	14.6 (8.9–20.4)	18.5 (15.4–21.4)	66.9 (58.8–75.0)		17.2 (10.8–23.6)	17.7 (10.8–23.6)	65.1 (58.8–71.3)	
11th	20.3 (16.3–24.3)	13.0 (7.0–18.9)	66.7 (59.4–74.1)		15.6 (9.6–21.6)	17.2 (11.0–23.5)	67.2 (58.4–76.0)	
12th	18.0 (13.3–22.7)	30.3 (25.9–34.7)	51.7 (45.5–57.9)		20.1 (14.1–26.1)	25.3 (19.0–31.6)	54.7 (47.3–62.0)	
**Sex**				0.4053				0. 0795
Male	13.9 (10.2–17.6)	19.1 (14.7–23.5)	67.0 (62.9–71.1)		15.2 (10.8–19.6)	16.2 (13.1–19.3)	68.6 (62.5–74.7)	
Female	16.1 (9.7–22.6)	16.0 (13.3–18.8)	67.9 (59.9–75.9)		18.5 (13.6–23.4)	19.5 (15.7–23.3)	62.0 (56.2–67.8)	
**Race**				0.8462				0.0402*
American Indian only	15.3 (7.8–22.7)	17.2 (14.1–20.3)	67.5 (61.3–73.8)		19.1 (13.7–24.5)	17.5 (13.5–21.4)	63.5 (56.6–70.4)	
Non-Hispanic White	13.6 (8.8–18.3)	17.9 (15.4–20.4)	68.5 (63.4–73.7)		12.9 (10.1–15.6)	18.3 (14.2–22.5)	68.8 (63.5–74.1)	
**Ethnicity**				0.2017				0.6554
Hispanic	20.2 (6.8–33.5)	21.3 (11.1–31.4)	58.6 (45.7–71.4)		18.0 (8.4–27.5)	20.6 (13.2–28.0)	61.5 (55.3–67.7)	
Non-Hispanic	14.6 (10.6–18.6)	17.6 (15.5–19.6)	68.1 (63.8–71.9)		16.8 (13.2–20.3)	17.6 (14.5–20.7)	65.4 (60.1–71.2)	
**Cigarette use**				< .0001*				< .0001*
Never	3.9 (1.1–6.7)	7.3 (6.0–8.5)	88.9 (85.3–92.5)		6.6 (4.0–9.2)	11.3 (8.7–13.9)	82.0 (78.0–86.1)	
Former	15.8 (8.9–22.7)	34.0 (25.5–42.5)	50.2 (40.8–59.6)		26.5 (20.8–32.1)	35.8 (31.0–40.6)	37.7 (31.3–44.2)	
Current	56.0 (38.5–73.5)	32.0 (15.4–48.6)	12.0 (5.2–18.7)		56.7 (46.8–66.7)	21.2 (14.7–27.8)	22.1 (14.5–29.6)	
**E-cigarette use**				< .0001*				< .0001*
Never	4.1 (2.0–6.2)	5.0 (2.3–7.8)	90.8 (87.8–93.9)		1.7 (0.4–2.9)	5.0 (3.5–6.5)	93.4 (91.0–95.9)	
Former	15.3 (4.9–25.7)	29.7 (16.3–43.0)	55.0 (40.1–70.0)		10.8 (4.5–17.1)	26.3 (21.7–30.9)	62.9 (54.3–71.5)	
Current	35.9 (19.1–52.6)	32.7 (15.3–50.1)	31.4 (25.8–37.1)		42.6 (35.4–49.9)	26.2 (18.3–34.2)	31.2 (25.4–36.9)	
**Smokeless tobacco use**				< .0001*				< .0001*
None in Past Mo	12.9 (9.9–15.9)	16.4 (12.2–20.5)	70.7 (64.7–76.8)		14.9 (11.0–18.7)	17.6 (14.5–20.9)	67.4 (62.1–72.8)	
Current	31.8 (16.8–46.9)	30.6 (13.5–47.7)	37.6 (30.1, 45.1)		42.0 (30.3–53.8)	19.2 (7.3–31.2)	38.7 (28.3–49.2)	
**Alcohol use**				< .0001*				< .0001*
Never	1.8 (0.2–3.5)	2.2 (0.2–4.2)	98.7 (93.2–98.7)		2.0 (0.7–3.4)	5.6 (2.8–8.30)	92.4 (88.9–95.9)	
Former	8.2 (3.8–12.6)	21.7 (15.4–28.0)	70.1 (62.3–77.9)		12.4 (7.0–17.8)	24.6 (19.2–30.0)	63.0 (56.1–70.0)	
Current	35.4 (27.3–43.5)	29.3 (23.3–35.3)	35.3 (31.0–39.5)		42.6 (36.5–48.7)	27.0 (19.9–34.0)	30.4 (30.0–35.9)	
**Binge drinking**				< .0001*				< .0001*
None in past Mo	7.1 (4.1–10.2)	13.4 (11.3–15.5)	79.4 (75.3–83.6)		8.6 (5.6–11.7)	14.9 (12.3–17.4)	76.5 (71.9–81.1)	
Current	44.9 (32.1–57.7)	28.4 (15.9–40.8)	26.6 (20.1–33.3)		49.1 (40.9–57.3)	30.2 (18.3–42.2)	20.7 (12.8–28.5)	
**Used illegal drugs**				< .0001*				< .0001*
Yes	46.1 (37.0–55.3)	25.4 (16.5–34.4)	28.5 (23.6–33.7)		32.6 (26.0–39.0)	31.1 (25.9–36.4)	36.3 (29.3–43.3)	
No	7.1 (5.0–9.2)	15.3 (10.7–20.0)	77.6 (72.0–83.1)		12.7 (8.6–16.8)	14.2 (10.3–17.9)	73.2 (66.8–79.5)	

^*^p value significant

### 2017

When we look at other substance use (cigarettes, e-cigarettes, smokeless tobacco, and alcohol), we see some strong associations. In 2017, among those who have never used cigarettes we see that only 3.9% (95% CI 1.1–6.7%) reported current use of cannabis, compared to current cigarette users where 56.0% (95% CI 38.5–73.5%) reported current use. Moreover, only 12.0% (95% CI 5.2–18.7%) of those who reported they were current cigarette users in 2017 also reported that they had never used cannabis, compared to a much larger 88.9% (95% CI 85.3–92.5%) of those who reported never trying cigarettes having never used cannabis (Table 2). We see an even more dramatic difference when looking at e-cigarette use in 2017 with 4.1% (95% CI 2.0–6.2%) of those never using e-cigarette were currently using cannabis compared to 35.9% (95% CI 19.1–52.6%) among those who were current e-cigarette users and 15.3% (95% CI 4.9–25.7%) among those who were former users of e-cigarettes (Table 2).

We see a similar rise in cannabis use in 2017 with smokeless tobacco use where 12.9% (95% CI 9.9–15.9%) of non-users using reporting current use of cannabis compared to 31.8% (95% CI 16.8–46.9%) of current smokeless tobacco users (Table 2). With alcohol use we see a similar pattern in 2017 with those who reported never using alcohol showing a very low rate of current cannabis use at 1.8% (95% CI 0.2–3.5%) compared to those with former or current use of alcohol reporting 8.2% (95% CI 3.8–12.6%) and 35.4% (95% CI 27.3–43.5%), respectively. Similarly, in 2017, among those who have reported NOT binge drinking in the last month, 7.1% (95% CI 4.1–10.2%) were current cannabis users compared to a larger 44.9% (95% CI 32.1–57.7%) reporting current cannabis use among those who currently binge drink (Table 2). Finally, in 2017, those who used illegal drugs were significantly more likely to be current users of cannabis at 46.1% (95% CI 37.0–55.3%) compared to the 7.1% (95% CI 5.0–9.2%) who never used illegal drugs but currently use cannabis.

### 2019

In 2019, among those who have never used cigarettes we see that only 6.6% (95% CI 4.0–9.2%) reported current use of cannabis, compared to current cigarette users where 56.7% (95% CI 46.8–66.7%) reported current use. Only 22.1% (95% CI 14.5–29.6%) of those who reported they were current cigarette users in 2019 also reported that they had never used cannabis, compared to a much larger 82.0% (95% CI 78.0–86.1%) of those who reported never trying cigarettes having never smoked cannabis (Table 2). A more noticeable difference is noted when looking at e-cigarette use in 2019 with 1.7% (95% CI 0.4–2.9%) of those never using e-cigarette were using cannabis compared to 42.6% (95% CI 35.4–49.9%) among those who currently are current e-cigarette users and 10.8% (95% CI 4.5–17.1%) among those who are former users of e-cigarettes.

Likewise, cannabis use in 2019 mirrors the trends in 2017 in smokeless tobacco use where 14.9% (95% CI 11.0–18.7%) of non-users using reporting current use of cannabis compared to 42.0% (95% CI 30.3–53.8%) of current smokeless tobacco users (Table 2). With alcohol use in 2019, those who reported never consuming alcohol showing a very low rate of cannabis use at 2.0% (95% CI 0.7–3.4%) compared to those who currently or formerly used alcohol, reporting 42.6% (95% CI 36.5–48.7%) and 12.4% (95% CI 7.0–17.8%), respectively. In 2019, among those who have reported NOT binge drinking in the last month, 8.6% (95% CI 5.6–11.7%) were current cannabis users compared to a larger 49.1% (95% CI 40.9–57.3%) reporting current cannabis use among those who currently binge drink (Table 2). Finally, in 2019, those who used illegal drugs were significantly more likely to be current users of cannabis at 32.6% (95% CI 26.0–39.0%) compared to those 12.7% (95% CI 8.6–16.8%) who reported never using illegal drugs.

### Multivariate Logistic Regression Modeling Results

When investigating each substance along with demographic predictors univariably against the students’ use of cannabis, predictors age, grade, cigarette use, e-cigarette use, smokeless tobacco use, alcohol use, binge drinking, and illegal drug use were all significant (*p* < 0.05). Year of survey (2017 vs 2019), student age, and race, however, were not significant (*p* > 0.05). There was high collinearity among student age and grade level, as expected, and age was the preferred predictor used in the final model (r > 0.70). Age was chosen to more closely compare students regardless of progression in school, but rather to investigate social differences by birth year. Evaluation of interaction terms identified that year did not significantly interact with the other terms in the model and univariably was insignificant, but the term was retained in the model for interpretation as it is the primary interest for this study. Cigarette use significantly interacted with e-cigarette use, while illegal drug use had significant interaction with binge drinking and alcohol use. With insufficient counts for these variables, interaction terms were dropped from the model.

In addition, due to small cell counts in several predictor variables, certain categories were combined to improve statistical power. Age categories were collapsed from the range of 12–17 to 15 or younger, 16, and 17 years or older. Cigarette use was reduced to compare ‘Ever’ users, which includes both ‘current’ and ‘former’ cigarette users. E-cigarette use was compared between current users and non-users which includes ‘former’ and ‘never’ users. Smokeless tobacco and binge drinking were compared between ‘non-users’ (those who did not use in the past month) and ‘current’ users. Also, alcohol use was reduced to report the differences between ‘current’ users compared ‘non-users’ consisting of ‘former’ and ‘never’ alcohol users.

Investigation into confounding identified behaviors of cigarette, e-cigarette, smokeless tobacco, alcohol, and illegal drug use as potential confounders. Additionally, the relationship between sex and cannabis use was confounded as the association in Table 2 is significant, but the model output in Table 3 lacks significance. The results of the Likelihood Ratio Test identified the full model containing predictors of age, sex, race, cigarette, e-cigarette, smokeless tobacco, and alcohol use along with binge drinking and illegal drug use as the most appropriate model (*p* < 0.0001).

**Table 3:** Results from multivariate logistic regression Cherokee Nation Youth Risk Behavior Survey, Cherokee Nation Reservation 2017 and 2019

Population characteristics	Current vs former			Current vs never			Former vs never		
	OR	95% CL	P-value	OR	95% CL	P-value	OR	95% CL	P-value
**Year of survey**									
2017									
2019	0.925	0.680–1.260	0.6151	1.275	0.925–1.757	0.1338	1.232	0.883–1.720	0.2143
**Age**									
< = 15 years old									
16 years old	0.983	0.767–1.260	0.8903	1.324	0.930–1.886	0.1166	1.271	0.931–1.735	0.1278
17 + years old	0.995	0.742–1.336	0.9753	1.486	1.019–2.166	0.0401*	1.332	0.968–1.833	0.0772
**Sex**									
Female	1.191	0.887–1.599	0.2390	1.436	1.029–2.004	0.0342*	1.140	0.836–1.554	0.4001
Male									
**Race**									
American Indian only	1.344	1.081–1.672	0.0090*	1.245	0.900–1.723	0.1804	0.918	0.670–1.259	0.5878
Non-Hispanic White									
**Cigarette use**									
Ever	0.657	0.421–1.026	0.0643	5.489	3.365–8.955	< 0.0001*	6.123	4.099–9.147	< 0.0001*
Never									
**E-cigarette use**									
Current	1.605	0.593–4.347	0.3436	3.088	1.939–4.918	< 0.0001*	2.246	1.286–3.925	0.0054*
Never + former									
**Smokeless Tobacco use**									
Any	1.427	0.433–4.702	0.5513	0.784	0.444–1.386	0.3943	0.630	0.305–1.302	0.2065
None in Last Mo									
**Alcohol use**									
Current	0.912	0.535–1.556	0.7306	2.001	1.211–3.305	0.0078*	1.842	1.159–2.927	0.0109*
Never + Former									
**Binge drinking**									
Current	2.711	1.055–6.967	0.0388*	1.756	0.897–3.440	0.0983	1.119	0.603–2.078	0.7150
None in past Mo									
**Illegal drug use**									
Yes	1.450	0.826–2.547	0.1903	3.308	2.356–4.645	< 0.0001	2.056	1.373–3.079	0.0008*
No									

^*^p value significant

### Demographic Predictors (Year, Age, Sex, and Race)

Among survey participants, the odds of current cannabis use compared to both former cannabis and never cannabis use did not significantly differ by year of survey, age, or sex (Table 3). After adjusting for other predictors, students aged 17 and older had 1.486-times the odds of being current cannabis users versus never cannabis users (*p* = 0.04, 95% CL 1.02–2.17). There was no significant difference between those aged 16 compared to those 15 and younger when investigating current versus never cannabis use (*p* = 0.116, Table 3). Similarly, females had 1.436-times greater odds of being current rather than never cannabis users compared to male students, after adjusting (*p* = 0.034, 95% CL 1.03–2.00).

Student odds of cannabis use, current versus never or former versus never, was not significantly related to reported race (Table 3). However, after adjusting for other factors, students identifying as AIAN had 1.344 times greater odds of being current cannabis users against former use compared to NHW students (*p* = 0.009, 95% CL 1.08–1.67). Race did not significantly differ when comparing current to never and former to never cannabis users (Table 3).

### Nicotine Consumption (Cigarette, E-Cigarette, and Smokeless Tobacco Use)

Cigarette, e-cigarette, and smokeless tobacco use were not significant predictors of current cannabis use when compared to former cannabis users (Table 3). Notably, after adjusting for other predictors, students who reported having ever smoked cigarettes had 5.489-times greater odds of being current cannabis users against never users and 6.123-times greater odds of being former cannabis users against never users, when compared to their non-smoking classmates (*p* < 0.0001, 95% CL 3.37–8.96; *p* < 0.001, 95% CL 4.10–9.15, respectively).

Similarly, after adjusting for other predictors, students who reported current use of e-cigarette products had 3.088-times greater odds of being current against never cannabis users and 2.246- times greater odds of being former cannabis users against never users, when compared to their never and former e-cig using classmates (*p* < 0.0001, 95% CL 1.94–4.92; *p* = 0.0053, 95% CL 1.29–3.93, respectively). There was no significant relationship between current, former, or never cannabis use based on students’ smokeless tobacco use in the past month (Table 3).

### Alcohol And Illegal Drug Use (Alcohol, Binge Drinking, and Illegal Drug Use)

Alcohol use and illegal drug use were not significant predictors of current cannabis use compared to former cannabis users (Table 3). However, after adjusting for other predictors, students who reported currently drinking alcohol on a regular basis had 2.001-times greater odds of being current compared to never cannabis users and 1.842- times greater odds of being former users compared to never cannabis users (*p* = 0.008, 95% CL 1.21–3.31; *p* = 0.011, 95% CL 1.16–2.93, respectively). Interestingly, after adjusting for other predictors, students who reported binge drinking in the past month had 2.711-times greater odds of being current against former cannabis users compared to students who have never participated in binge drinking (*p* = 0.0388, 95% CL 1.06–6.97). But students who were current binge drinkers did not have higher odds of being current against never or former against never cannabis users (Table 3). Illegal drug use was not significantly related to current cannabis use when compared to former cannabis using students. Nevertheless, after adjusting for other predictors, students who reported currently using any illicit drugs surveyed had 3.308-times greater odds of being current against never cannabis users and 2.056-times greater odds of being former against never cannabis users compared to those who had never used any of the illegal drugs in question (*p* < 0.0001, 95% CL 2.36–4.65; *p* = 0.0008, 95% CL 1.37–3.08, respectively).

## Discussion

Among youth in the Cherokee Nation Reservation, there was no change in cannabis use between 2017 and 2019 despite the 2018 legalization of medical cannabis in Oklahoma. There were, however, differences in cannabis use by demographic variables as well as by other substance use. We saw differences in age with those aged 17 and older at increased odds of cannabis use than those 15 years or younger. Additionally, when compared to males, females’ odds were higher for current use compared to those who never used cannabis. AIAN students showed higher odds of current compared to former cannabis use compared to NHW students. There were no differences based on ethnicity. Notably, we also saw differences when looking at other substance use except smokeless tobacco. Both ‘ever’ cigarette users and ‘current’ e-cigarettes users were at increased the odd of current and former cannabis use against never use. Alcohol use increased the odds of both former and cannabis use among current uses compared to never and formers users. Those who reported binge drinking alcohol or using illegal drugs showed an increased in the odds of both former and current cannabis use against never use.

The legalization of medical cannabis in Oklahoma did not seem to increase the rate of use among youth in high school in the Cherokee Nation Reservation. This finding contributes to the somewhat confusing literature on access to and use of cannabis that suggests decreased use with legalization of cannabis [10, 29–32, 42–50]. An ecological study in Montana showed the relation between number of medical cannabis cards and youth use was associated with voting for recreation use (representing social norms) rather than living in an area with more medical cannabis cards [43]. This study, however, did not account for dispensary density. A nearly ten-year-old study (2014) from California showed that physical availability (shown through road dispensary density) of medical cannabis use and frequency of use [44]. Additionally, a study from Los Angeles California showed that among youth more medical cannabis dispensaries was associated with more frequent use in the past month [51]. Another earlier study in Colorado suggests that while no increase in risk of use, there was an increased risk of abuse or dependence on cannabis [45]. The Healthy Kids Colorado Survey results showed no change in use with the legalization of recreational cannabis use in Colorado; however, there was an increased perceived ease of access [42]. Finally, in a national study looking at cannabis use in the past year using a national panel, showed risk for use higher among states with recreational use compared to states without legal cannabis use [34]. Our results suggest that increased access through medical legalization did not increase use of cannabis among a population of AIAN and NHW students in the Cherokee Nation Reservation. Future studies should include newer (2021) YRBSS data to see if the expected trend occurs after time. Our results contribute to the literature by describing a specific population, AIAN and NHW in the Cherokee Nation Reservation. How, after the first year of medical cannabis legalization, trends of cannabis use did not increase. In short, this study supports the idea that medical cannabis legalization does not show an increase in youth prevalence of cannabis use, despite increased availability.

In this study, we show a significantly increased odds of currently using cannabis among women compared to men. After adjusting for other factors, students identifying as AIAN had 1.4 times greater odds of being current cannabis users against former use compared to NHW students. This agrees with most other research with AIAN adults or youth included [23, 24, 27, 28]. The factors associated with cannabis use in the Cherokee Nation Reservation included age, grade, cigarette use, e-cigarette use, alcohol use, and the use of illegal drugs but not smokeless tobacco. The association of age and grade were expected with an overall trend toward increasing rates as either increase is commonly seen [47]. There is strong longitudinal data showing the relationship between cigarette [52, 53] as well as e-cigarettes [52, 54–59] with future cannabis use as well as evidence of cannabis use leading to cigarette use [15, 60]. A 2013 systematic review and meta-analysis of the association between e-cigarettes among youth showed a significant increase in the odds of current or past cannabis use in youth [61]. Additionally, they found the odds of past or current cannabis use among youth who used e-cigarettes was 3.5 times higher than for those who denied e-cigarette use with younger youth (ages 12 to 17) showing a stronger association [61]. The trajectory of poly-use is still unclear [62]. Alcohol use was clearly associated with cannabis use, but it is difficult to define the multiple pathways in this relationship. A 2017 review of the co-use of alcohol and cannabis use reported that while there is clear evidence of co-use and of poorer outcome for those using both substances, there needs to be more longitudinal studies to better understand the relationship [63]. In this study, we show a significantly increased odds of currently using cannabis among women compared to men. While historically males use was higher the differences have been decreasing with this being one of the first studies showing higher female use compared to males [48, 64]. Our results show that AIAN and NHW students in the Cherokee Nation Reservation did show a small but insignificant use when looking at current vs former uses (OR 1.4 p-value 0.0344). In short, this study supports the idea that AIAN youth have slightly higher (but not substantially higher) rates of use at least compared to NHW students.

The strengths of this study are many. The CNYRBS is based on a standard well validated, high reliability survey [65–67]. It is administered by an experienced team of public health staff within Cherokee Nation who have about 15 years of conducting the YRBS, weighted and cleaned with YRBS standard protocols [41]. The analysis team included collaboration between Cherokee Nation and University of Oklahoma employees working together. Another strength of this study was the clear delineation of the legislative change. The implementation was well publicized with a clear licensing method. These data represent an underrepresented population AIAN living in rural environments.

There are still a few limitations to this study. First, there were insufficient counts of students who identified as Hispanic or responded positively to any of the illegal drug use questions, interaction terms had to be dropped from the model. Second, due to small cell counts in several predictor variables, certain categories were combined to improve statistical power. Third, the CNYRBS was completed in spring of 2019 so there was limited implementation time between the first licensing for medical cannabis in Oklahoma on August 25, 2018, and the survey dates.

## Conclusion

Despite the legalization of medical cannabis in Oklahoma in 2018, cannabis use remained unchanged among youth in the Cherokee Nation Reservation between 2017 and 2019. However, variations in use were observed based on age, gender, other substance use, with older individuals, females, and those using cigarettes, e-cigarettes, alcohol, or illegal drugs having higher odds of cannabis use.

### Supplementary Information

Below is the link to the electronic supplementary material.

Supplementary file1 (DOCX 39 kb)

## Data Availability

Restrictions apply to the availability of these data, which are owned by the Cherokee Nation, and are not publicly available. Data are, however, available with the explicit permission of the Cherokee Nation.

## Abbreviations

AI: American Indian
AIAN: American Indian Alaskan native
CNYRBS: Cherokee nation youth risk behavior survey
NHW: Non-Hispanic white
NSDUH: National survey on drug use and health
NESARC: National epidemiologic survey on alcohol and related conditions
OMMA: Oklahoma medical marijuana authority
YRBS: Youth risk behavior survey
