# Supplementary material for: Investigating Cannabis-Use Among Students Attending High Schools Within the Cherokee Nation Reservation 2017 and 2019
Source: J Community Health. 2023 Dec 8;49(3):402–14. doi: 10.1007/s10900-023-01304-7 (PMC10981585; doi:10.1007/s10900-023-01304-7)
Supplement: Supplementary file 1 — Supplementary file1 (DOCX 39 kb) [file 10900_2023_1304_MOESM1_ESM.docx]

| Supplemental Information S1: Cherokee Nation Youth Risk Behavior Survey questions used | | |
| --- | --- | --- |
| **Cannabis Use (Primary Outcome)** |  |  |
| “The next 3 questions ask about cannabis use. Cannabis also is called pot, weed, or cannabis.” | “During your life, how many times have you used cannabis?” | 0 times  1 or 2 times  3 to 9 times  10 to 19 times  20 to 39 times  40 to 99 times  100 or more times |
|  | “How old were you when you tried cannabis for the first time?” | I have never tried cannabis  8 years old or younger  9 or 10 years old  11 or 12 years old  13 or 14 years old  15 or 16 years old  17 years old or older |
|  | “During the past 30 days, how many times did you use cannabis?” | 0 times  1 or 2 times  3 to 9 times  10 to 19 times  20 to 39 times  40 or more times |
|  |  |  |
| **Cigarette Smoking** |  |  |
| “The next 4 questions ask about cigarette smoking.” | Have you ever tried cigarette smoking, even one or two puffs?” | Yes  No |
|  | “How old were you when you first smoked a cigarette, even one or two puffs?” | I have never smoked a cigarette, not even one or two puffs  8 years old or younger  9 or 10 years old  11 or 12 years old  13 or 14 years old  15 or 16 years old  17 years old or older |
|  |  |  |
| **E-cigarette Use** |  |  |
| “The next 3 questions ask about electronic vapor products, such as blu, NJOY, Vuse, MarkTen, Logic, Vapin Plus, eGo, and Halo. Electronic vapor products include ecigarettes, e-cigars, e-pipes, vape pipes, vaping pens, e-hookahs, and hookah pens.” | “Have you ever used an electronic vapor product?” | Yes  No |
|  | “During the past 30 days, on how many days did you use an electronic vapor product?” | 0 times  1 or 2 times  3 to 9 times  10 to 19 times  20 to 39 times  40 or more times |
|  | “During the past 30 days, on how many days did you smoke cigarettes?” | 0 days  1 or 2 days  3 to 5 days  6 to 9 days  10 to 19 days  20 to 29 days  All 30 days |
|  |  |  |
| **Smokeless Tobacco** |  |  |
|  | “During the past 30 days, on how many days did you use chewing tobacco, snuff, dip, snus, or dissolvable tobacco products, such as Redman, Levi Garrett, Beechnut, Skoal, Skoal Bandits, Copenhagen, Camel Snus, Marlboro Snus, General Snus, Ariva, Stonewall, or Camel Orbs? (Do not count any electronic vapor products.)?” | 0 days  1 or 2 days  3 to 5 days  6 to 9 days  10 to 19 days  20 to 29 days  All 30 days |
|  |  |  |
| **Alcohol** |  |  |
| “The next 4 questions ask about drinking alcohol. This includes drinking beer, wine, wine coolers, and liquor such as rum, gin, vodka, or whiskey. For these questions, drinking alcohol does not include drinking a few sips of wine for religious purposes.” | “During your life, on how many days have you had at least one drink of alcohol?” | 0 days  1 or 2 days  3 to 5 days  6 to 9 days  10 to 19 days  20 to 29 days  All 30 days |
|  | “How old were you when you had your first drink of alcohol other than a few sips?” | I have never tried alcohol  8 years old or younger  9 or 10 years old  11 or 12 years old  13 or 14 years old  15 or 16 years old  17 years old or older |
|  | “During the past 30 days, on how many days did you have at least one drink of alcohol?” | 0 days  1 or 2 days  3 to 5 days  6 to 9 days  10 to 19 days  20 to 29 days  All 30 days |
|  |  |  |
| **Binge Drinking** |  |  |
| The next 2 questions ask about how many drinks of alcohol you have had in a row, that is, within a couple of hours. For the first question, the number of drinks you need to think about is different for female students and male students.” | “During the past 30 days, on how many days did you have 4 or more drinks of alcohol in a row (if you are female) or 5 or more drinks of alcohol in a row (if you are male)?” | 0 days  1 or 2 days  3 to 5 days  6 to 9 days  10 to 19 days  20 or more days |
|  |  |  |
| **Illegal Drugs** |  |  |
| The next 6 questions ask about other drugs. | “During your life, how many times have you used any form of cocaine, including powder, crack, or freebase?” | 0 times,  1 or 2 times  3 to 9 times  10 to 19 times  20 to 39 times  40 or more times |
|  | “During your life, how many times have you sniffed glue, breathed the contents of aerosol spray cans, or inhaled any paints or sprays to get high?” | 0 times,  1 or 2 times  3 to 9 times  10 to 19 times  20 to 39 times  40 or more times |
|  | “During your life, how many times have you used heroin (also called smack, junk, or China White)?”; | 0 times,  1 or 2 times  3 to 9 times  10 to 19 times  20 to 39 times  40 or more times |
|  | “During your life, how many times have you used methamphetamines (also called speed, crystal meth, crank, ice, or meth)?”; | 0 times,  1 or 2 times  3 to 9 times  10 to 19 times  20 to 39 times  40 or more times |
|  | “During your life, how many times have you used ecstasy (also called MDMA or Molly)?” | 0 times,  1 or 2 times  3 to 9 times  10 to 19 times  20 to 39 times  40 or more times |
|  | During your life, how many times have you used a needle to inject any illegal drug into your body?” | 0 times  1 time  2 or more times |
| Investigating Cannabis-Use among Students attending High Schools within the Cherokee Nation Reservation 2017 and 2019: An epidemiology study  Journal of Community Health  Janis E. Campbell, Ph.D.,^1^ Sixia Chen, Ph.D.,^1^ Anna Bailey, MPH^1^, Andrea Blair, Ph.D.^2^ Ashley L. Comiford, Dr.P.H.,^3^  1. Department of Biostatistics and Epidemiology, Hudson College of Public Health, University of Oklahoma Health Sciences Center, Oklahoma City, OK 73104, USA.  2. Cherokee Nation Public Health, 1325 East Boone Street, Tahlequah, OK 74464, USA.  3. Cherokee Nation Health Services, 19600 East Ross Road, Tahlequah, OK 74464, USA.  Corresponding Author: [janis-campbell@ouhsc.edu](mailto:janis-campbell@ouhsc.edu) | | |
